# Probing mental representations of space through sketch mapping: a scoping review

**DOI:** 10.1186/s41235-025-00667-w

**Published:** 2025-09-03

**Authors:** M. Simonet, C. Vater, C. Abati, S. Zhong, P. Mavros, A. Schwering, M. Raubal, C. Hölscher, J. Krukar

**Affiliations:** 1 Chair of Cognitive Science, ETH Zürich, Zürich, Switzerland; 2 Institute of Sport Science, University of Bern, Bern, Switzerland; 3 Institute of Cartography and Geoinformation, ETH Zürich, Zürich, Switzerland; 4 i3 (UMR 9217), CNRS, Télécom Paris, Institut Polytechnique de Paris, Palaiseau, France; 5 Institute for Geoinformatics, University of Münster, Münster, Germany

**Keywords:** Scoping review, Mental representation of space, Spatial cognition, Sketch map

## Abstract

Cognitive maps are mental representations of space essential for guiding spatial behavior. To assess the properties of these cognitive maps, sketch mapping has been widely used as a research tool in spatial cognition research. This scoping review aims to provide a comprehensive overview of the methodologies and the cognitive processes concerning the externalization of cognitive maps through sketch mapping. Following the PRISMA-ScR checklist (Tricco et al. in Ann Intern Med 169(7):467–473, 2018, https://doi.org/10.7326/M18-0850), a comprehensive search from five electronic databases was performed with predefined combinations of keywords. Twenty-four articles were selected and analyzed, covering a wide range of methods: traditional pen-and-paper sketching (*n* = 18 studies); combination of pen-and-paper and digital sketching (*n* = 1); exclusively digital sketching (*n* = 4); and digital VR sketching (*n* = 1). With regard to the formation of cognitive maps in environmental scale spaces, studies employed either direct experience or virtual experience of unfamiliar environments, videos, maps, or retrieval from own memory. This review highlights the inherent conflict between sketch maps’ advantages in capturing knowledge in less structured experimental protocols and researchers’ need for structured quantification of their quality, as well as the underused diversity of media through which sketch maps can be produced for appropriate scenarios. We encourage researchers to (a) increase the precision in reporting the cognitive processes being investigated with sketch maps (e.g., short-term vs. long-term memory), (b) rely on established data analysis methods instead of developing custom measures for each study, and (c) consider alternative media beyond pen and paper when more suitable to the experimental context.

**Supplementary Information:**

The online version contains supplementary material available at 10.1186/s41235-025-00667-w.

## Significance statement

Mental representations of space, known as cognitive maps, are essential for finding our way and engaging with the world around us. This research examines how cognitive maps are externalized through sketch mapping, a widely used tool in spatial cognition research. Sketch maps—informal drawings based on an individual's memory of an environment—have long been used to explore human spatial understanding and decision-making.

The study addresses a real-world problem: understanding how people mentally organize complex spaces like buildings and cities, which is essential for fields like urban planning, architecture, or wayfinding systems in transportation hubs among others.

By synthesizing findings from 24 studies, this review identifies key cognitive processes—such as spatial memory, problem-solving, and spatial reasoning—that are probed with the use of sketch maps across literature. Furthermore, it highlights the emerging divergence of methods used to collect sketch maps, ranging from traditional pen-and-paper techniques to digital tools and virtual reality opening new possibilities. Despite this increasing variety, underrepresentation of some issues persists, e.g., representing environments with vertical components, such as multi-floor buildings. The review emphasizes the need for clearly defining the specific cognitive processes targeted in sketch map studies, such as distinguishing between short-term and long-term memory. Additionally, we recommend using established analysis methods and considering alternative media beyond pen and paper when better aligned with the experimental design.

**Supplementary Information:**

The online version contains supplementary material available at 10.1186/s41235-025-00667-w.

## Introduction

Everyday navigation involves a multitude of diverse decisions, including choosing paths, estimating distance, and identifying landmarks (Dalton et al., 2019; Golledge, 1999; Passini, 1981), rendering it a complex and demanding task. During navigation, people both create and rely on mental representations of their surroundings, known as cognitive maps (Portugali, 1996; Tolman, 1948; Tversky, 1993). These representations include, for example, information about object locations and paths to an object.

Mental representations of space contain distortions and deviations from geometric properties of space (such as scale, distance, and angle) (Tversky, 1993) and large individual differences can explain how well people can learn and remember an environment (Ishikawa & Montello, 2006). Understanding how people perceive and manage this spatial information has significant implications for tasks like navigating in complex buildings (Hölscher et al., 2009; Morag et al., 2024), piloting a plane (Sutton et al., 2014), driving a cab in a big city center (Maguire et al., 2006), exercising in an urban park (Cai et al., 2021), or finding ways to leave a building in fire emergency situations (Meng & Zhang, 2014).

Perceptual processes enable humans to gather information about the environment when navigating. The visual scene processing is important to create mental representations of space (Malcolm et al., 2016). Following successful visual scene processing, spatial attention and memory processes play a crucial role in developing knowledge of the environment. This is important for integrating, encoding, and storing spatial information from multiple scenes (Albert et al., 1999; Czajkowski et al., 2014). Neuroscientific data have also confirmed this, by showing that an experience-dependent memory trace is formed in a specific brain structure, i.e., the retrosplenial cortex, that processes and stores spatial information (Czajkowski et al., 2014). Such a cognitive map allows people to accumulate, code, store, recall, and manipulate the obtained spatial information about the environment (Stea, 2017). Research on spatial perception has presented different cognitive mechanisms for horizontal and vertical dimensions (Churches et al., 2017). Horizontal biases tend to depend more on space-based processes, while vertical biases are more influenced by object-based mechanisms (Churches et al., 2017). Evidence suggests that the perception of vertical space diverges from that of horizontal space, with greater distortions and overestimations occurring in vertical judgments (Dixon & Proffitt, 2002; Prytz & Scerbo, 2012).

The cognitive map can be externalized through sketch maps that make the spatial knowledge of an environment visible and accessible to others (Epstein et al., 2017). Sketch mapping requires both cognitive and physiological processes (Frith & Law, 1995) and allows to assess the mental representation of space (Appleyard, 1970; Krukar et al., 2018; Lynch, 1996). It is a complex activity that relies on higher-order cognitive processes such as creative problem-solving, critical thinking, and working memory (Lane, 2018; Teixeira De Almeida et al., 2023).

So far, studies have assessed spatial knowledge using two-dimensional (2D) sketches with mainly pen-and-paper approaches (Kim et al., 2022). Attempting to represent three-dimensional (3D) mental models (e.g., from multi-story buildings) on a flat 2D sheet of paper leads to distortions, scaling problems, and loss of information (Kim et al., 2022; Tversky, 2003). For example, traditional 2D sketching methods might fail to capture the complexity of environments with vertical dimensions.

This scoping review aims to provide a comprehensive overview of (a) cognitive processes involved in the process of externalizing a cognitive map into a sketch map and (b) methods employed for studying this. The review focuses on research involving spaces categorized under the'environmental'scale of Montello’s classification (Montello, 1993), excluding those falling into the'figural','vista', and'geographical'scales.'Environmental'scale refers to spaces that are too vast to be directly comprehended without extensive movement, typically requiring information integration over prolonged periods. Spaces such as buildings, neighborhoods, and cities fall within the environmental scale. We selected this particular category because we are interested in the externalization of mental representations of space, which are constructed through the navigation and memory-driven integration of views experienced separately and understood through multiple perspectives gained during locomotion. Focusing on a specific scale is important for the cognitive perspective of the review, as previous research has shown that spatial memory formed in environmental space differs from that formed in vista space (Meilinger et al., 2016). For instance, it has been shown that memorizing space at the environmental scale is influenced by the distance traveled along a path and the sequential order in which objects are learned (Meilinger et al., 2016).

### Objectives

The scoping review aims to identify current knowledge on the externalization of cognitive maps of environmental scale spaces through sketch mapping. The specific research questions are:

1. What are the methods (tools, experimental protocol) used in sketch mapping research?
2. Which cognitive processes involved in understanding the environment and externalizing it into sketch maps are described in the literature?
3. What are the emerging research directions, opportunities, and challenges

## Methods

### Protocol and registration

The Preferred Reporting Items for Systematic Reviews and Meta-analysis Protocols extension for Scoping Review (PRISMA-ScR; Tricco et al., 2018) was used to enable replication of the review. A protocol was written before the literature search and approved by all the authors but was not registered.

### Information sources and search strategy

Before identifying relevant journal articles, three authors (SM, VC, and KJ) approved the search keywords and the eligibility criteria. We included all published articles that involved adult participants, were written in English, and had been published until the start of the literature search (15th of December, 2023). Systematic reviews were also considered. In accordance with Montello's classification (Montello, 1993), our scoping review solely included research involving environments categorized under the ‘environmental’ scale. Consequently, even small environments with spatial boundaries that necessitate movement to explore what lies beyond those boundaries were included (Meilinger et al., 2016). Peer review was not an inclusion criterion. However, non-peer-reviewed articles included in the review (*n* = 4 conference papers) were assessed for quality. Table 1 summarizes the eligibility criteria.

**Table 1:** Eligibility criteria based on study population, environment, sketching method, and types of evidence

	Inclusion	Exclusion
Population	► Adults	► Non-human participants ► Children, elderly (> 65 years old)
Environmental scale	► ‘Environmental’ scales based on Montello's classification, e.g., city, neighborhood, building	► ‘Figural’, ‘vista’, and ‘geographical’ scales
Types of evidence	► Published articles ► Full-text articles ► Preprints ► Full-text conference proceedings ► Articles written in English ► Publication timeframe: until the start of the search	► Note ► Editorial articles ► Abstracts or posters ► Articles for which we cannot obtain the full text ► Articles that are not written in English ► Dissertations/Theses ► Book

The search strategy was developed and drafted by the authors *SM* and *VC* and was refined through discussion with all authors. To collect the relevant literature, PubMed, Web of Science Core Collection, Scopus, Dimensions, and Embase were searched using the following combination of keywords: (“cognitive map” *OR* “spatial representation*” *OR* “mental model*” *OR*"cognitive collage"*OR*"mental representation*"*OR*"navigation"*OR*"wayfinding"*OR*"spatial memor*"*OR*"cognitive process*") *AND*"sketch*".

### Selection of sources of evidence

The study selection was conducted through three stages: (1) examining the titles, (2) reading the abstracts, and (3) reviewing the full text. Two separate raters independently processed these stages and evaluated the articles according to the eligibility criteria. If there were any differences in their assessments, they were resolved through discussion with a third rater to ensure the quality of the search. Prior to study selection, two raters conducted a training exercise using a random sample of 50 titles and abstracts from the initial search to ensure consistency in the screening process. The raters independently assessed whether each paper should be included in the review and then compared their results. In cases of discrepancy, a third rater provided feedback and guidance to maintain the quality of the selection process. After this training exercise, both raters independently screened the titles and abstracts for inclusion. The identical training exercise including 10 articles was performed again for reviewing the full-text articles. In cases of discrepancy, a third rater provided feedback and guidance to maintain the quality of the selection process. After that, the two raters independently evaluated the full-text articles for inclusion.

### Data item

Based on our eligibility criteria, a standardized data abstraction form was developed a priori of screening full-text articles. Two raters independently abstracted the data from the full-text articles, filled in the table developed for this review, and then compared their results. A third rater resolved discrepancies. The extracted data were organized into tables (please see Appendix 1 and Appendix 2), where each row corresponds to individual articles, the columns represent the classification, and the cells contain the pertinent information. Regarding the taxonomy of the new table, the categorization was informed by key references in the field (Hegarty et al., 2006; Krukar et al., 2023). Notably, after the review process, we identified three papers in which no explicit experimental phase of *space acquisition* was present, as the tasks involved required participants to create or design spatial layouts rather than learn them. In Appendix 2, we have labeled the *space acquisition* phase in these cases as *Design task*, adopting the terminology used by the original authors.

#### Cognitive processes identification protocol

**Fig. 1:**
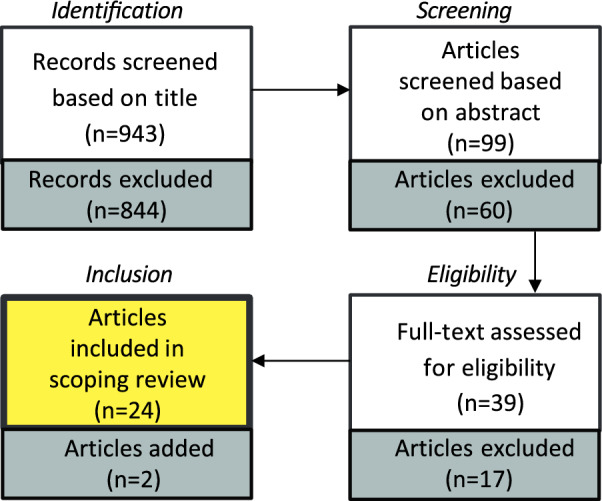
Flow of information through the different phases of the scoping review

While all the included studies involve participants performing cognitive tasks and assessing cognitive functioning, many do not directly address these aspects. According to Harvey (2019), the primary components of cognitive functioning include sensation, perception, motor skills and construction, attention and concentration, memory, processing speed, language/verbal skills, as well as executive functioning, including reasoning and problem-solving as subdomains. To investigate the cognitive processes addressed in the selected papers, we developed the following protocol. Based on the “Domains of cognitive functioning” presented by Harvey (2019), we searched for specific keywords within the *Methods*, *Results*, and *Discussion* sections of each paper. The keywords included: “Sensat”, “Percept”, “Motor skill”, “Motor ability”, “Attent”, “Memory”, “Recall”, “Executive funct”, “Reasoning”, “Problem solv” or “problem-solv”, and “Processing speed”. We excluded the *Introduction* sections from our search, as they typically reference other studies rather than focusing on the specific cognitive processes investigated in the current paper (Fig. 1).

**Fig. 2:**
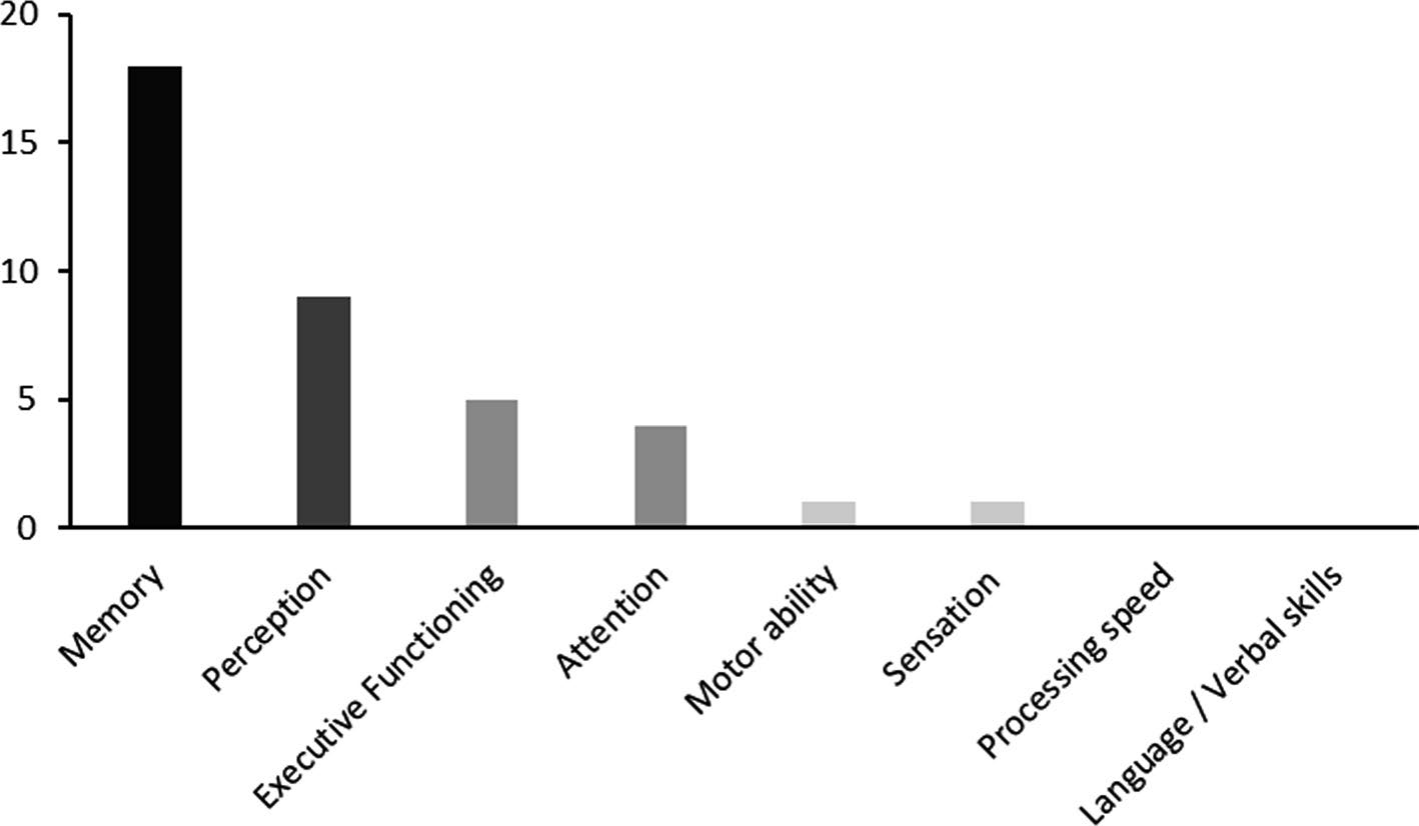
Number of papers investigating each identified domain of cognition by Harvey et al. (2019)

For each occurrence of these keywords, we documented them, and the numbers of papers investigating each cognitive processes can be found in Fig. 2.

#### Link between space acquisition and the medium used for sketching

A contingency table was created to examine the relationship between space acquisition modes and sketching media (see Table 2). Given the small frequency in some categories, Fisher's exact test was applied in R (version 2023.06.2 + 561), as it is appropriate for categorical data with low expected frequencies. To ensure statistical validity and avoid computational limitations, certain space acquisition modes and sketching media were merged based on conceptual similarity.

**Table 2:** Contingency table of the relationship between space acquisition modes and sketching media

Contingency Table			
Space acquisition	Medium		
	Digital	Pen and paper	Total
Direct experience	1	4	5
Past experience	3	8	11
Virtual	1	5	6
Design	0	3	3
Total	5	20	25

Each cell represents the number of papers corresponding to a specific combination of these variables

### Synthesis of results

**Fig. 3:**
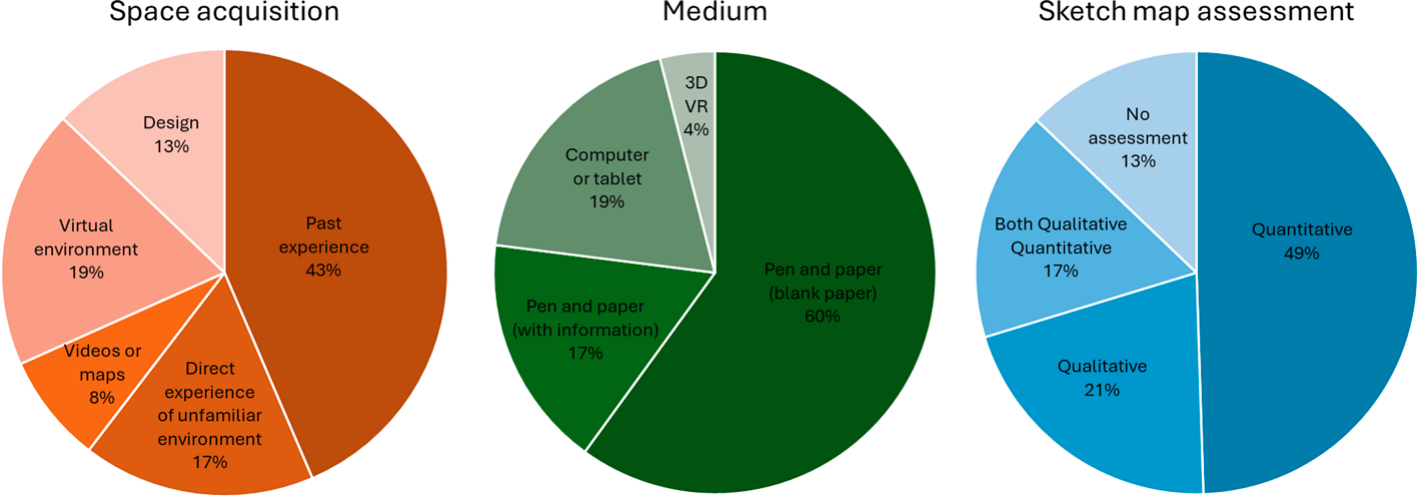
Methodologies used in the studies as a percentage of the total number of studies. Note: “Space acquisition” refers to how participants acquire knowledge of the spatial environment before sketching. “Medium” refers to the tools used for sketching. “Sketch map assessment” refers to the type of analyses used to assess the sketch maps

In alignment with the objectives of this scoping review, the data synthesis (*Appendix 1 and Appendix 2, *Table 3, Fig. 2) focused on providing information about the methods and tasks used in the sketching literature, as well as the cognitive processes investigated. Figure 3 provides a visual representation of the methods used for space acquisition, the media utilized, and the sketch map assessments.

**Table 3:** Synthesis of the correlations between sketch map metrics with other measures of spatial knowledge

	Sketch map metrics	Other measures of spatial knowledge	Statistics
Billinghurst and Weghorst (1995)	Map goodness score	Knowing where everything is in the Virtual World	Virtual Valley: *r* = 0.635, *p* < 0.05
			Neighborhood: *r* = 0.405, *p* < 0.05
			Cloudlands: *r* = 193, *p* > 0.05
	Map goodness score	Orientation in the Virtual World	Virtual Valley: *r* = 0.738, *p* < 0.05
			Neighborhood: *r* = 0.524, *p* < 0.05
			Cloudlands: *r* = 290, *p* > 0.05
	Map goodness ranking	Ease of interaction	Virtual Valley: *r* = 0.882, *p* < 0.05
			Neighborhood: *p* > 0.05
			Cloudlands: *p* > 0.05
		Ease of navigation	Virtual Valley: *r* = 0.865, *p* < 0.05
			Neighborhood: *p* > 0.05
			Cloudlands: *p* > 0.05
		Ease of movement	Virtual Valley: *r* = 0.814, *p* < 0.05
			Neighborhood: *p* > 0.05
			Cloudlands: *p* > 0.05
		Ease of use of Data Globe	Virtual Valley: *r* = 0.645, *p* < 0.05
			Neighborhood: *p* > 0.05
			Cloudlands: *p* > 0.05
Dong et al. (2022)	Sketch map performance	Direction and Distance	Negative weak correlation*
	Sketch map performance	Distance estimation	Strong positive correlation
Gehrke et al. (2018)	Sketch map usefulness	Perspective taking skills	*r* = 0.39, *p* < 0.05
	Sketch map usefulness	Experienced realism inside the virtual environment	*r* = 0.41, *p* < 0.05
Jaeger et al. (2023)	Number of target details	Within-pointing	*r*(49) = −0.35, *p* = 0.01
	Number of target details	Between-pointing error	*r*(49) = −0.29, *p* = 0.04
	Number of target details	Model building	*r*(49) = 0.20, *p* = 0.17
	Route details score	Silcton performance measures	*r*(49) = −0.37, *p* < 0.01
		Between-pointing error	*r*(49) = −0.52, *p* < 0.001
		Model building	*r*(49) = 0.37, *p* < 0.01
Wang and Schwering (2015)	Sketch map accuracy	Participants'sketching skill, artistic talent, cartographic knowledge, occupation and geo-science background	No correlation
	Sketch map accuracy & Sketch map completeness	Familiarity (frequency of visits to study area)	Higher familiarity led to more buildings drawn and more complete street networks with higher accuracy (no analysis provided)

The asterisk (*) indicates that all Pearson correlation values are available in Dong et al. (2022) in Figure 8 C of the paper, as there were too many to include here

## Results

### Selection of sources of evidence

In total, 1866 papers were found (PubMed *n* = 117; Web of Science *n* = 520; Scopus *n* = 761; Embase *n* = 96; Dimensions *n* = 372). After removing duplicates (*n* = 923), we identified and screened 943 articles for eligibility. Ninety-nine articles were selected after screening the titles. The abstracts of the 99 articles were explored, and 39 articles were found to be eligible based on our exclusion/inclusion criteria. Finally, and after the full-text assessment phase, 22 were retained for this scoping review. The main reasons for excluding the papers were: no use of sketching, environments not corresponding to Montello’s environmental scale, not written in English, not human, or inclusion of children. At the end of the process, we added two additional articles that were not found with the search keywords but were of relevance for our review based on co-authors’ comments and cross-referencing other papers.^1^ Figure 1 illustrates the flow of information through the different phases of the scoping review.

### Characteristics of sources of evidence

The main characteristics of the selected studies are presented in Appendix 1. This table summarizes the study details (authors in alphabetical order and year of publication, type of publication), the goal of the study, the population characteristics (sample size, gender), the tasks, measures, and main results. Appendix 2 summarizes how space is acquired, the environment to be sketched, the medium used to sketch, and the sketch maps assessment. Table 3 lists the papers in which correlations between sketch maps and spatial knowledge have been assessed.

### Results of individual sources of evidence

The results of the selected studies can be found in Appendix 1 and Appendix 2, as well as in Table 3.

### Synthesis of results

The studies encompassed publication dates from 1970 to 2023. Among the 24 studies included in the analysis, fifteen were journal articles and nine were conference papers. The sample sizes ranged from 6 to 460 participants. Seventeen studies provided detailed demographic information regarding the gender distribution of participants. The literature review identified various methods used for sketching (details in Appendix 2). The most common method was traditional pen-and-paper sketching (*n* = 18 papers), followed by digital sketching (*n* = 4 papers), a combination of pen-and-paper and digital sketching (*n* = 1 paper), and digital sketching in virtual reality (VR) (*n* = 1 paper). In terms of the environment, most studies used environments with no vertical components (*n* = 19) like neighborhoods, and five studies used environments with a vertical component such as buildings. The perceptual-cognitive processes discussed in the selected articles include sensation, perception, (spatial) memory, problem-solving, attention, (spatial) reasoning, and motor ability. This covers six domains out of eight identified by Harvey (2019), excluding processing speed and language/verbal skills.

Regarding the methodologies used, the results show diverse technological setups for spatial information acquisition and sketch mapping. Figure 3 provides an overview (in percentage of all the studies) of the tasks used to acquire the spatial information, referring to “space acquisition”, the medium used to sketch, and the sketch map assessment.

#### Link between space acquisition and the medium used for sketching

There was no evidence of a significant association between the mode of space acquisition and the medium used for sketching (*p* = 1), indicating that the two variables are independent.

## Discussion

This scoping review aims to provide a comprehensive overview of the current literature on the externalization of mental representations of space through sketch mapping, focusing on three central research questions: What are the methods (tools, experimental protocol) used in sketch mapping research? Which cognitive processes involved in understanding the environment and externalizing it into sketch maps are described in the literature? And what are the emerging research directions, opportunities, and challenges? The review highlights the cognitive functions that are crucial for the acquisition and externalization of spatial knowledge, examining how these processes are represented in the literature. Additionally, it offers a description of the various tools and methods used in sketch mapping research. By synthesizing existing findings, this review not only enhances our understanding of the cognitive processes and methods involved in sketch mapping but also identifies critical gaps in knowledge, pointing to promising areas for future investigation.

### Methods (tools, experimental protocol) used in sketch mapping research

Researchers employed various approaches to studying the externalization of mental representation of space. In this section, based on the results extracted from this review, we aim to address the following sub-questions:

- What types of analyses are commonly applied in sketch mapping research?
- How do sketch map metrics correlate with other measures of spatial knowledge?
- What sketching media are used, and are they appropriate for sketching the environment?

#### S*ketch map analysis: great diversity and inherent conflicts*

The methods for generating and assessing sketch maps varied significantly among studies. These differences highlight the complexity of the subject and underscore the limited replicability and generalizability of the findings.

Overall, studies employed quantitative, qualitative, or a combination of both approaches to analyze sketch maps. Quantitative analyses include measures such as completeness (Billinghurst & Weghorst, 1995; Huynh et al., 2010; Imani & Tabaeian, 2012), sketch quality scoring (Jaeger et al., 2023), sketch map distortion (Erem, 2021; Imani & Tabaeian, 2012), or other measures such as the number of participants remembering sketch map elements, distances between landmarks or the buffer methods (Lopez & Bosco, 2022; Okamoto et al., 2005). Qualitative analyses of sketch maps include measures such as map goodness (Billinghurst & Weghorst, 1995), map types (Anacta et al., 2014; Appleyard, 1970; Huynh et al., 2008; Wang & Schwering, 2015), and usefulness (Gehrke et al., 2018).

One significant limitation of quantitative methods is that they overemphasize metric accuracy of information contained within sketch maps, punishing the participant's score for mismatch of angles and distances between spatial information in the sketch and in the corresponding ground-truth maps. Since we know that sketch maps are inherently generalized, inaccurate, and incomplete (Manivannan et al., 2022; Schwering et al., 2022; Wang & Schwering, 2015), quantitative methods might misrepresent the value of participant's knowledge that—even if only approximately metric (Ishikawa & Montello, 2006)—might be sufficient to successfully guide behavior. An inherent conflict identified by this review is that researchers predominantly use sketch maps in unstructured ways—naturalistic environments sketched on blank paper—along their inherent ambiguity and variability (see Fig. 3). While more structured and controlled environments, such as video-based or VR-based scenarios, or tools providing digital scaffolding, could yield clearer, more structured data, researchers continue to favor unstructured sketch mapping. Ironically, these unstructured sketches are then assessed using predominantly quantitative methods biased toward objective quantification. This may reflect a broader disciplinary tendency within environmental psychology to undervalue qualitative data (Lloyd & Gifford, 2024). This tension highlights an ongoing methodological challenge: how to balance spontaneous, naturalistic context of sketch maps with the demands of quantitative rigor and objectivity.

A bridge between quantitative analyses and more permissive (yet difficult to operationalize and replicate) qualitative analyses is the approach of formal analysis of qualitative spatial relations. Qualitative spatial relations refer to structured descriptions of spatial configurations, such as relative positions (e.g., “A is north of B”, “landmark C proceeds landmark D along street 1”, or “landmark E is inside region X”), that can be quantitatively scored (Chipofya et al., 2011). These representations capture essential aspects of spatial layouts without relying on exact metric precision, while still allowing for objective evaluation. As such, qualitative spatial relations serve as a bridge between qualitative assessments, like map goodness, and more quantitative measures, such as completeness: while not restricted by metric accuracy in Euclidean space, they still yield objective and comparable data.

Finally, three studies did not assess sketch mapping performance, as their tasks focused on creative design (Bilda & Demirkan, 2003; Brösamle & Hölscher, 2018; Yuan et al., 2018). These studies emphasized the process of sketch generation rather than evaluating or scoring the resulting sketches.

#### The correlation of sketch map metrics with other spatial knowledge measures: present but unexplained

In addition to assessing the quality or the completeness of sketch maps, several studies have attempted to establish correlations between sketch mapping and spatial knowledge. For instance, Billinghurst and Weghorst (1995) found significant correlations between map goodness and measures of spatial knowledge and sense of orientation. The correlation between map goodness score and sense of orientation indicates that individuals who are more skilled at orienting themselves in space may produce more accurate and detailed sketch maps. Similarly, Gehrke et al. (2018) found a significant correlation between sketch map usefulness and the Perspective Taking and Spatial Orientation Test, suggesting that higher spatial abilities are linked to higher sketch map usefulness scores. Another study examined whether more accurate sketch maps correlate with better route or survey knowledge (Jaeger et al., 2023). To explore this, the authors examined the relationship between sketch map measures and performance in pointing and model building tasks. Generally, participants who included more target buildings in their drawings made fewer errors in their within- and between-route pointing judgments. Dong et al.'s (2022) analysis showed a weak relationship between estimation errors in distance and direction and sketch map performance. Poorer performance in estimating distance or direction was associated with lower sketch map performance. Finally, Wang and Schwering (2015) showed that familiarity, measured by the frequency of visits to the areas, influenced both accuracy and completeness of sketch maps. Participants who visited the areas daily included more buildings and drew more complete street networks with greater accuracy than those who visited only monthly. Overall, these findings show that sketch maps effectively represent environmental knowledge and serve as reliable indicators of spatial knowledge and navigational performance.

It seems plausible to assume that sketch maps are a more valid tool for capturing spatial knowledge for (a) some environment types and (b) some users. However, it remains unknown for which exactly. Our literature review demonstrates that answering this question is currently hindered by the diversity of methods with which sketch maps are analyzed and the diversity of alternative spatial knowledge measures against which they are validated. Each study identified in our review that analyzed such a correlation (see Table 3) did so for a *unique* combination of variables. This is problematic because researchers deciding on the use of sketch maps for their specific experimental use case currently have to rely on their own intuition and have no evidence base to guide or justify this choice.

#### Appropriateness of sketching medium: emerging diversity with underused potential

While traditional paper–pencil methods dominate the field, some studies integrated digital sketching tools such as a drawing panel (Dong et al., 2022), a tablet PC combined with a wireless pencil (Huynh et al., 2008, 2010; Tu Huynh & Doherty, 2007), or 3D VR sketching tool (Gehrke et al., 2018).

We investigated whether a relationship exists between the mode of space acquisition (categorized as past experience, direct experience, videos/maps, virtual environment, and others) and the medium used for sketching (pen and blank paper, pen and paper with information, digital sketching, 3D VR sketching).

Our results do not show a direct link between the mode of space acquisition and the medium used for sketching. This suggests that there is no clear consensus in the literature regarding the ideal sketching medium for a given mode of space acquisition and, consequently, for spatial externalization. This finding highlights the need for further investigation into the factors influencing the choice of space acquisition methods and the medium used for sketching. If a clear relationship between these two experimental choices could be established, it would enable researchers to formulate methodological recommendations for future studies in sketch mapping. More research on this is necessary since technological advancement has made it possible to draw sketch maps with an increasing diversity of media. For example, one common scenario currently includes exploring space in virtual reality and subsequently drawing a sketch map on a physical paper (Orellana & Al-Sayed, 2013). One relevant emerging research question is, therefore, whether breaking the presence of virtual reality experiments affects the type and quality of paper-based sketch maps, as opposed to drawing them directly inside VR. An unexplored mirrored use case is whether in some real-world scenarios it could be beneficial to produce sketch maps in AR/VR mode.

### Cognitive processes involved in understanding one’s environment and externalizing it through sketch maps

The included studies describe several cognitive functions critical to understanding and externalizing the environment into sketch maps. These cognitive functions encompass perception (9 studies) and memory (18 studies), reasoning (4 studies), attention (4 studies), problem-solving (1 study), sensation (1 study) and motor ability (1 study). In the included studies, perception and memory were the most prominent functions. We divided this section into two parts: cognitive processes during spatial knowledge acquisition and cognitive processes during sketch mapping.

#### Cognitive processes during spatial knowledge acquisition

Navigating and understanding space relies on one's ability to effectively process and store spatial information. Perception, attention, and memory are key to building mental representations of space. For instance, accurate distance perception influences navigation efficiency by impacting the estimation of time and speed (Dong et al., 2022). Individual cognitive functions and environmental context also significantly shape wayfinding and spatial perception (Imani & Tabaeian, 2012). Additionally, spatial memory is crucial for encoding, storing, and retrieving environmental knowledge, involving landmark anchoring and route integration strategies (Shi et al., 2020; Siegel & White, 1975). Factors such as conflicting virtual information can impair spatial memory (Liu et al., 2024), and visual attention directly affects the quality of spatial memory formed during exploration (Rautenbach et al., 2015). Furthermore, the format of spatial acquisition—whether 2D, 3D, or VR—has been shown to influence the quality of acquired spatial memories, with 3D environments typically yielding superior memory outcomes (Ye et al., 2021).

The way in which space is learned—whether through long-term, repeated experiences (such as daily navigation), or through short-term, one-time navigation tasks—may engage different cognitive processes (e.g., long-term vs short-term spatial memory). Therefore, implementing experimental protocols that would directly compare these conditions, e.g., by sketching familiar environment *versus* short exploration of a new environment, represents an open gap for future research.

#### Cognitive processes during sketch mapping

A second gap is the lack of consensus on isolating and capturing cognitive processes in sketch mapping studies. Our results indicate that memory is extensively reported (18 studies), i.e., most researchers see it as central to sketch map externalization. However, perception and attention (“input” processes that feed the accumulation of knowledge) are only discussed in studies explicitly dealing with initial space acquisition scenarios (e.g., Dong et al., 2022; Imani & Tabaeian, 2012). Reasoning and problem-solving processes are heavily underrepresented as they appear almost exclusively in design contexts (Bilda & Demirkan, 2003; Yuan et al., 2018). Furthermore, only few studies differentiate between short-term (immediate recall after exploration) and long-term (familiar environments) memory processes. This general treatment makes it difficult to precisely operationalize memory in sketch mapping studies, creating ambiguity about whether researchers measure long-term knowledge or short-term recall and how different forms of input contribute to different types of memory. It can be thus suggested that although sketch maps are used as a measure of spatial memory, they are treated as a relatively crude measure. The potential of this method to differentiate more specific cognitive processes informing different types of memory remains underutilized.

### Emerging research directions, opportunities, and challenges

An emerging mode of sketch mapping is VR or AR-based 3D drawing (Gehrke et al., 2018; Xiao et al., 2024). In the study by Gehrke et al. (2018), participants performed digital VR sketching using a computer mouse. By pressing the left mouse button, a red sphere appeared in the VR goggles, tracking the position of their right hand. This allowed participants to draw a red line by moving their hand through space. Interestingly, a covariation was observed between the feeling of presence experienced during exploration and the accuracy of the resulting sketch maps. This suggests that participants with higher immersion scores may have retained a more precise mental representation of the environment, enabling them to produce better sketches. In the context of growing popularity of VR for spatial cognition studies, the relation between presence and spatial knowledge acquisition is a key issue (Creem-Regehr et al., 2022). An emerging yet understudied research question is how the presence impacts one's ability to externalize this knowledge through VR/AR sketching tools.

Such sketching tools are particularly valuable for drawing environments that have an important vertical component (Kim et al., 2022), like flight paths (Wang et al., 2024) or multi-layer buildings (Xiao et al., 2024), as participants struggle with representing vertical relations consistently across the whole drawing (Zhong & Kozhevnikov, 2016). As an example, Xiao et al. (2024) showed that a method combining 2D surface sketching with mid-air sketching for producing 3D sketch maps significantly reduced distortions in drawings of multi-layered buildings. This highlights that AR and VR methods might open a range of new possibilities for the use of sketch mapping across a more diverse set of spatial scenarios, tasks, and environment types. Although navigating vertical dimensions occurs less frequently than horizontal movement, it requires critical decision-making, including determining when and where to change levels and maintaining an awareness of one’s position relative to vertical space (e.g., being above or below a target location) (Gath-Morad et al., 2021; Hölscher et al., 2006; Sohn et al., 2022). In this sense, vertical navigation in multi-floor buildings is not simply a contextual shift, such as moving from Floor A (represented by a 2D map) via an elevator to Floor B (another 2D map). For instance, exiting a metro station often demands knowing which side of the street the exit leads to, necessitating a mental representation of vertical spatial relationships.

There is also a visible absence of objective measures to assess the sketching process. For example, neuroimaging methods could contribute to understanding of the dynamic interactions between sketching and cognitive processes (Gehrke et al., 2018; Gramann et al., 2014) but have been hindered by movement-induced artifacts and the physical constraints of imaging techniques. One concept that is of clear interest but had been studied predominantly with subjective ratings is cognitive load during sketching across tools and environmental contexts (Liu et al., 2022; Naismith et al., 2015). Event-related potentials from electroencephalography and micro-saccade analysis using eye-tracking offer a promising alternative for the near future (Keskin et al., 2018; Teixeira De Almeida et al., 2023).

## Conclusion

In conclusion, this scoping review identified 24 studies exploring the externalization of cognitive maps through sketch mapping. The review highlights a significant diversity in the methodologies employed across studies, including variations in tasks, experimental designs, and sketching assessments. Additionally, our results revealed that the primary cognitive processes investigated in this field are memory and perception. However, these processes have yet to be thoroughly examined using more objective methods, such as identifying neural correlates underlying memory and perception during sketch mapping—an important direction for future research. Overall, this work underscores the inherent conflict between sketch maps’ advantages in capturing knowledge in less structured experimental protocols and researchers’ need for structured quantification of their quality, as well as the underused diversity of media through which sketch maps can be produced for appropriate scenarios. This manuscript opens promising avenues for future spatial cognition research, particularly in improving spatial representations and navigational aids to enhance individuals' ability to comprehend and navigate their environments effectively.

## Supplementary Information

Additional file 1.

Additional file 2.

## Data Availability

Not applicable.

## Footnotes

1 Blades (1990): While the title contains"sketch maps", the abstract lacks other key terms from our search strategy. The paper was not identified due to the restrictive use of AND"sketch*". Using OR"sketch*"broadened the search but led to too many irrelevant results. Appleyard (1970): The title lacks search terms like"sketch","cognitive map", or"spatial representation". The term"sketch"appears only once in the manuscript (not in the title, nor the abstract), which explains why it was not found.
